# Hydroxypropyl Methylcellulose and Gum Arabic Composite Edible Coatings Amended with Geraniol to Control Postharvest Brown Rot and Maintain Quality of Cold-Stored Plums

**DOI:** 10.3390/foods12152978

**Published:** 2023-08-07

**Authors:** Zahra Sadat Asgarian, Lluís Palou, Ricardo Felipe Lima de Souza, Paloma G. Quintanilla, Verònica Taberner, Rouhollah Karimi, María Bernardita Pérez-Gago

**Affiliations:** 1 Grapevine Production and Genetic Improvement Department, Iranian Grape and Raisin Institute, Malayer University, Malayer 65719-95863, Iran; z.s.asgarian@gmail.com (Z.S.A.); r.karimi@malayeru.ac.ir (R.K.); 2 Centre de Tecnologia Postcollita (CTP), Institut Valencià d’Investigacions Agràries (IVIA), Montcada, 46113 València, Spain; palou_llu@gva.es (L.P.); lima_ric@gva.es (R.F.L.d.S.); quintanilla_pal.evntl@gva.es (P.G.Q.); taberner_ver@gva.es (V.T.); 3 Vicerrectorat d’Investigació, Universitat Politècnica de València (UPV), Camí de Vera, s/n, 46022 València, Spain; 4 Department of Landscape Engineering, Faculty of Agriculture, Malayer University, Malayer 65719-95863, Iran

**Keywords:** stone fruit, *Monilinia fructicola*, postharvest quality, antifungal edible coatings, cold storage

## Abstract

In this study, the effect of hydroxypropyl methylcellulose (HPMC) and gum Arabic (GA) edible coatings amended with 0.2% geraniol (GE) were evaluated for the control of brown rot, caused by *Monilinia fructicola*, on artificially inoculated plums (*Prunus salicina* Lindl., cv. Angeleno) stored for 5 weeks at 1 °C. Brown rot is the most important pre- and postharvest fungal disease of stone fruits, causing severe economic losses worldwide. Geraniol is an important constituent of many essential oils that can be obtained as a byproduct from different industrial procedures, such as those of the juice industry. Fruit postharvest quality was also evaluated after 5 and 8 weeks of storage at 1 °C, followed by 3 days at 7 °C plus 5 days at 20 °C, simulating packinghouse, transport, and retail shelf-life conditions, respectively. HPMC coatings containing 0.2% GE reduced the incidence and severity of brown rot by 37.5 and 64.8%, respectively, compared to uncoated fruit after 5 weeks of storage at 1 °C. HPMC-coated plums, with and without GE, showed the highest level of firmness, the lowest change in external peel color parameters (L*, a*, b*, C*, hue), and the lowest flesh bleeding compared to uncoated control and GA-coated samples throughout the entire storage period, which correlated with a higher gas barrier of these coatings without negatively affecting sensory quality. Furthermore, the HPMC-0.2% GE coating provided the highest gloss to coated plums, showing the potential of this coating as a safe and environmentally friendly alternative to conventional fungicides and waxes for brown rot control and quality maintenance of cold-stored plums.

## 1. Introduction

Plum (*Prunus salicina* Lindl.) is an important summer crop worldwide. Plum fruit are highly appreciated for their nutritional and organoleptic properties, but several factors considerably limit their storability and shelf life. Among them, chilling injury (CI) causing internal breakdown and bleeding and postharvest decay caused by pathogenic fungi are the most important [1]. In general, low-temperature storage at 0–1 °C is recommended to maintain postharvest plum quality, while prolonged exposure to temperatures in the range 3–8 °C can cause CI, especially in the case of highly sensitive cultivars [2,3]. Conversely, storage at higher temperatures can contribute to the development of fungal infections and consequently increase the incidence of postharvest diseases, leading to fruit losses of up to 50% when no effective postharvest fungicides are applied [4].

Brown rot caused by *Monilinia* spp. (syn.: *Monilia* spp.) is one of the most important postharvest diseases of stone fruits in general and plums in particular [5]. Brown rot can be successfully controlled by pre- and postharvest treatments with effective fungicides [6]. However, prolonged and extensive use of postharvest chemical fungicides has raised alerts and legal restrictions on their use due to the problems related to chemical residues on produce and in the environment and the proliferation of fungicide-resistant strains. Therefore, various postharvest technologies of different nature have been tested to replace synthetic fungicides to control *Monilinia* spp. and maintain fruit quality [7]. Among them, antifungal edible coatings have become a promising approach to reduce or even replace the use of synthetic fungicides in fruit and extend postharvest shelf life [8,9]. These coatings, based on food-grade biodegradable materials such as polysaccharides, proteins, and lipids, alone or in combination, form a thin layer on the surface of the fruit that act as a barrier against moisture and gas exchange between the fruit and the environment, reducing weight loss and general metabolism, which translates into delayed ripening and senescence [10]. The incorporation into coating matrix emulsions of ingredients with antifungal character, such as biocontrol agents, food preservatives, and low-toxicity compounds classified as generally recognized as safe (GRAS) (e.g., essential oils, plant extracts, natural volatiles) can impart antifungal functionality to the coatings [11,12].

Although the ability of edible coatings based on starch [13,14,15,16], *Aloe vera* [17], chitosan [18,19,20], pectin [21], gum Arabic [22,23], carboxymethylcellulose [8,21], or hydroxypropyl methylcellulose (HPMC) [24] to reduce moisture loss and maintain texture and overall quality in plums has been documented in recent works, to our knowledge, only three studies have investigated the capacity of edible coating formulations to reduce postharvest plum decay. Thus, gum Arabic (GA) coatings containing oregano or rosemary essential oils inhibited the mycelial growth and spore germination of *Rhizopus stolonifer* in artificially inoculated plums [23]. In a study by Karaca et al. [6], different compounds classified as food additives or GRAS (generally recognized as safe) substances, mainly mineral and organic acid salts, were evaluated as antifungal ingredients of HPMC-based edible coatings to reduce brown rot on plums artificially inoculated with *Monilinia fructicola*, and potassium sorbate and paraben salts were the most effective. In a more recent work, More et al. [15] reported that taro starch–casein coatings enriched with pomegranate peel extract controlled microbial growth caused by natural infections on coated fruit. Therefore, HPMC and GA are appropriate hydrocolloids for developing novel antifungal edible coatings and assessing their efficacy not only to control decay caused by *Monilinia* spp. or other important postharvest pathogens in plums, but also to regulate the fruit’s physiological behavior and maintain fruit quality during storage. This double beneficial action by a single postharvest treatment may enormously contribute to reducing the economic losses of fruit growers and traders in important plum producing countries such as Spain and Iran.

The production of high-value functionalized molecules extracted from abundant, cheap, and renewable feedstocks such as plants and food industry byproducts is an area of great interest. Preliminary work in our laboratory has proven the efficacy of the volatile compound geraniol in inhibiting the mycelial growth of *M. fructicola* in in vitro studies. Geraniol is an acyclic monoterpene alcohol released from many plant organs, such as flowers, herbs, seeds, and fruits [25,26]. Beyond its contribution to flavor, geraniol is a common component of many essential oils with antimicrobial properties found in different fruits, vegetables, and herbs, including tea, lemongrass, lavender, lemons, plums, and grapes, among others. Some of these essential oils and geraniol can be obtained as a byproduct (raw materials, fruit peels, etc.) from different industrial procedures such as, for example, the juice extraction for fruit- and plant-based beverages [27,28]. Furthermore, several studies have also reported the effective in vitro antifungal activity of geraniol against *Botrytis cinerea*, *M. fructicola* [29], and *Penicillium digitatum* [30]. Therefore, this work aimed to investigate the effect of HPMC- and GA-based coatings containing geraniol as antifungal agent on (i) the control of *M. fructicola* on inoculated plums during cold storage at 1 °C and (ii) the physicochemical and sensory quality of plums cold-stored at 1 °C for up to 8 weeks followed by 3 days at 7 °C, simulating transportation conditions, plus 7 days at 20 °C, simulating retail handling conditions.

## 2. Materials and Methods

### 2.1. Coating Materials and Ingredients

Coating emulsions consisted of hydroxypropyl methylcellulose (HPMC; Methocel E19, Dow Chemical Co., Midland, MI, USA) or gum Arabic (GA; Instantgum AA, Nexira, Rouen, France), as the hydrophilic phase, and beeswax (BW; Fomesa Fruitech S.L., Valencia, Spain) as the lipidic phase. Minor components of the coatings were glycerol (Panreac Química S.L., Barcelona, Catalonia, Spain), used as a plasticizer, and stearic acid (Panreac Química, S.L.) and sucroester fatty acid (SE; P-1570, Cymit Química S.L., Barcelona, Catalonia, Spain), used as emulsifiers. Geraniol (GE, Sigma-Aldrich, St. Louis, MO, USA) was the antifungal ingredient added to the coating matrixes.

### 2.2. Preparation of Antifungal Edible Coatings

To prepare the HPMC emulsions, HPMC (2%, *w*/*w*) was dispersed in hot water at 90 °C and later hydrated at 20 °C to achieve complete solubilization. Next, BW was added at 0.8% (*w*/*w*), glycerol was added as a plasticizer at a HPMC:glycerol ratio of 3:1 (*w*/*w*), and stearic acid and SE were added as emulsifiers at 0.2% and 0.3% (*w*/*w*), respectively. GA emulsions were prepared from an aqueous solution of 3% (*w*/*w*) GA heated to 40 °C. Next, 0.25% SE was added as emulsifier, keeping the stirring until the solution became clear. Finally, BW at 0.5% and glycerol at 1.0% were added to the solution. The HPMC- and GA-mixtures were heated above 90 °C to melt the lipid and homogenized using a high-shear mixer (Ultra-Turrax model T25, IKA-Werke, Steufen, Germany) for 1 min at 17,000 rpm followed by 3 min at 22,000 rpm. The emulsions were then placed in an ice-water bath until their temperature reached below 25 °C. The incorporation of GE was performed in emulsions at room temperature with further homogenization for 2 min at 16,000 rpm. The stability of the prepared emulsions (with and without GE) was evaluated by placing them in volumetric tubes in a temperature-controlled cabinet at 25 °C and visually assessing the existence of phase separation after 24 h. The emulsions were further characterized by measuring viscosity and pH using the equipment described by Karaka et al. [6]. The viscosity values obtained for HPMC and GA emulsions were of 89.5 and 3.5 mPa.s, respectively, and the pH values for these emulsions were of 5.1 and 4.6, respectively.

### 2.3. Fruit Samples

Japanese plums (*Prunus salicina* Lindl.) cv. Angeleno were harvested in commercial orchards in Pobla del Duc (Valencia, Spain), transferred the same day to the IVIA research facilities, and cold stored at 1 °C and 90% relative humidity (RH) until use in the experiments, not later than 1–3 days after reception. Fruit were selected based on color, size, and absence of external damage and disinfected with a 0.5% sodium hypochlorite solution (diluted bleach) for 4 min, followed by rinsing in tap water and air-drying at room temperature (23–25 °C).

### 2.4. Effect of Coatings on Brown Rot Control

#### 2.4.1. Fungal Pathogen and Inoculum Preparation

*Monilinia fructicola* (G. Winter) honey strain MeCV-2 used in this study belongs to the IVIA CTP collection of postharvest pathogens. This strain was isolated from a rotten peach fruit from a stone fruit packinghouse in Carlet (Valencia, Spain) and, after isolation and identification, it was selected among other isolates due to its aggressiveness and uniform behavior. The strain is deposited and available with the accession number CECT 21161 at the Spanish Type Culture Collection (CECT, University of Valencia, Valencia Spain). Before the experiment, the isolate was grown on potato dextrose agar medium (PDA, Sigma-Aldrich Chemie, Steinheim, Germany) in Petri dishes at 25 °C for 7–14 days. High-density conidial suspensions of spores were prepared in Tween 80 (0.05% *w*/*v*; Panreac-Química S.A., Barcelona, Catalonia, Spain) in sterile water. After passing through two layers of cheesecloth, the density of the suspension was measured with a hemacytometer and diluted with sterile water to obtain an exact inoculum density of 1 × 10^3^ spores/mL [6].

#### 2.4.2. Curative Activity of Antifungal Coatings

Selected and surface disinfected plums were randomly divided into five groups, which corresponded to four coating treatments (HPMC and GA coatings with and without GE) and one water-dipped treatment as uncoated control. Before coating, plums were artificially inoculated in the equator using a stainless steel rod with a probe tip 1 mm wide and 2 mm in length, previously immersed into a spore suspension containing 1 × 10^3^ spores/mL of *M. fructicola*. After incubation at 20 °C for 24 h to simulate the real commercial time between infection production and postharvest treatment in the packinghouse, fruit were individually coated with 300 µL of coating material, rubbing with gloved hands to mimic the application of coating machines in the industry [6]. Coated fruit were placed on a mesh screen to air dry at room temperature. Inoculated but uncoated fruit were used as controls. Coated and control plums were placed on plastic trays on corrugated cartons and then stored for up to 5 weeks at 1 °C and 90% RH, plus 3 days at 20 °C to simulate retail conditions. Every week, brown rot incidence was determined as the percentage of infected fruit and brown rot severity as the diameter of the lesion (mm). Each treatment was applied to 4 replicates of 20 fruit each. Results obtained at week 5 are presented.

### 2.5. Effect of Coatings on Plum Fruit Quality

Selected and surface-disinfected plums, randomly distributed into lots of 120 fruit per treatment, were manually and individually coated as described previously. Control samples were immersed for 15 s in tap water at 20 °C. Fruit quality attributes described below were evaluated at harvest and after 5 and 8 weeks of storage at 1 °C and 90 ± 5% RH, followed by 3 days at 7 °C and 7 days at 20 °C and 90 ± 5% RH, simulating storage at the packinghouse, transportation, and retail handling conditions, respectively (60 fruit were used per storage period).

#### 2.5.1. Plum Weight Loss

Weight loss of 24 fruit per treatment was calculated by weighting individual fruit at the beginning and the end of each storage period. Results were expressed as the percentage of initial weight loss.

#### 2.5.2. Plum Flesh Firmness

Plum flesh firmness was measured in 24 plums per treatment using the methodology described by Gunaydin et al. [24].

#### 2.5.3. Plum Peel Color

External surface color of 24 plums per treatment was measured with a colorimeter (Model CR-400, Minolata, Tokyo, Japan) using the CIELAB (Commission Internationale de l’Eclairage) color parameters L*, a*, b*, Chroma (C*), and hue angle (h). Color of each fruit was measured at three different locations.

#### 2.5.4. Plum Juice Quality

Fruit internal quality attributes were determined in plum juice. Three replicates per treatment of 10 plums each were juiced with an industrial juicer (LOMI model 4, Barcelona, Catalonia, Spain), filtered through cheesecloth, and analyzed. Total soluble solid content (SSC, °Brix), titratable acidity (TA, g/L malic acid), and maturity index (MI, ratio SSC/TA) were determined as previously described [24].

#### 2.5.5. Ethanol and Acetaldehyde Content

The content of these fermentation volatiles was analyzed using headspace gas chromatography according to Gunaydin et al. [24]. Results are expressed as mg/L.

#### 2.5.6. Plum Physiological Disorders

The most important physiological disorder affecting plum flesh is CI, the main symptoms of which are flesh browning and/or bleeding [31]. At each evaluation date, sample plums were cut in half and evaluated visually in the mesocarp and around the pit. Internal browning was measured with a scale ranging from 1 (none) to 6 (severe, >75% of the flesh surface). Flesh bleeding was scored with values from 1 (none) to 3 (severe, >50% of the flesh surface). Results are presented as an average index obtained from 3 replicates of 10 fruit each.

#### 2.5.7. Plum Sensory Analysis

The regulation ISO 8586:2012 [32] was used as a general guidance for sensory analysis. At the end of each storage period, 18 semi-trained panelists evaluated the coded samples from each treatment served at room temperature under white illumination in individual booths in a sensory evaluation room. The evaluated sensory attributes were the following: overall flavor (with scores from 1 = very poor to 9 = optimum), off-flavor (from 1 = absence to 5 = high presence), firmness (from 1 = very soft to 5 = very firm, at the touch of the fruit), and visual fruit external aspect (score 1 = bad, 2 = acceptable, and 3 = good). Panelists were also asked to visually rank the five treatments from lowest to highest gloss, assigning, e.g., score = 1 to the least glossy treatment and score = 5 to the glossiest treatment. Then, the ranks from the 18 panelists were summed to present the results. The greatest sum of ranks indicates the glossiest treatment.

### 2.6. Statistical Analysis

Analysis of variance (ANOVA) and Fisher’s protected least significant difference test (LSD, *p* < 0.05) were performed to analyze the data from the different experiments, using the software Statgraphics Centurion XVII (Statgraphics Technologies Inc., The Plains, VA, USA). For disease incidence data, the ANOVA was applied to the arcsine-transformed data in order to assure the homogeneity of variances. The sum of ranks obtained from the plum gloss evaluation was analyzed using the Friedman test (*p* < 0.05). Presented data are means ± standard errors in every case.

## 3. Result and Discussion

### 3.1. Effect of Coatings on Brown Rot Development

**Figure 1:**
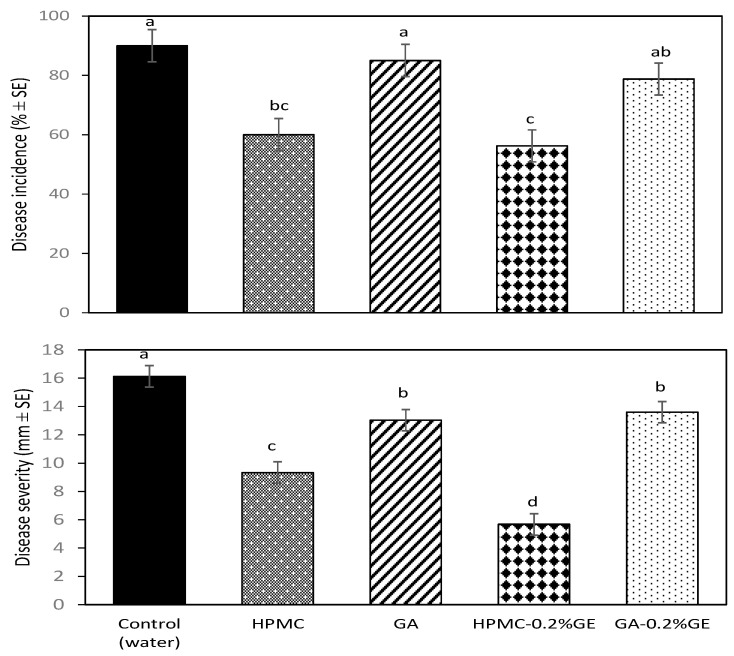
Incidence and severity of brown rot on ‘Angeleno’ plums artificially inoculated with *Monilinia fructicola*, uncoated (control) or coated 24 h later with hydroxypropyl methylcellulose (HPMC)-or gum Arabic (GA)-based edible composite coatings amended with geraniol (GE), and stored for 5 weeks at 1 °C and 90% RH. Means with different letters are significantly different according to Fisher’s protected LSD test (*p* < 0.05).

Both brown rot incidence and severity increased, although slowly, during storage at 1 °C. Figure 1 shows disease development after 5 weeks of storage at 1 °C, when disease incidence on uncoated control plums was higher than 90%. HPMC-based coatings significantly reduced brown rot incidence and severity compared to uncoated samples. The incorporation of GE to the HPMC-based coatings did not increase disease incidence reduction on coated plums, with an average reduction of 35% for both HPMC and HPMC-0.2% GE coatings. However, the HPMC-0.2% GE coating was more effective to reduce brown rot severity than the coating without GE, with reductions compared to control samples of 65 and 42%, respectively. On the contrary, GA-based coatings, alone or in combination with GE, did not significantly reduce disease incidence and had only a slight effect on reducing plum disease severity (*p* < 0.05). When plums were transferred to 20 °C after cold storage, disease development increased, but disease severity on fruit coated with HPMC-GE was still reduced by 40% compared to control plums.

The incorporation of essential oils or volatile compounds such as GE into edible coatings has been widely reported to reduce postharvest losses in fruits and vegetables. However, to our knowledge, the use of biopolymer-based edible coatings formulated with an antimicrobial volatile compound to control brown rot on plums inoculated with *M. fructicola* is reported here for the first time. Thus, for example, HPMC coatings incorporated with oregano and bergamot essential oils were evaluated for their suitability to reduce the survival of total microbial cells on plum surface, showing 1 and 2 log reductions on fruit stored at 5 °C for 1 month and at 23 °C for 14 days, respectively [8]. Similarly, carnauba wax nanoemulsions containing lemongrass essential oil were developed to inhibit the growth of *Salmonella typhimurium* and *Escherichia coli* O157:H7 on plums during storage at 4 and 25 °C, with initial inhibitions of up to 2.8 log [33]. Andrade et al. [23] evaluated the effect of GA coatings amended with oregano or rosemary essential oils to control Rhizopus soft rot on plums during 12 days of storage at room temperature and 24 days at 12 °C, obtaining incidence reductions of around 85 and 100%, respectively.

In our work, the HPMC coating formulated with 0.2% GE significantly reduced the incidence and severity of brown rot compared to uncoated plums, while the same concentration of GE incorporated to the GA-based coating did not affect disease development. GE is a monoterpenoid present in many essential oils that has proven different in vitro antifungal effects against a range of fruit pathogens, such as *Alternaria alternata* [34], *Rhizopus stolonifer* [35], *Colletotrichum* spp. [36], *Botrytis cinerea*, and *M. fructicola* [29]. Although the mode of action of monoterpenoids is not completely understood, variations in the antifungal activity of the oil components seemed to depend on solubility as well as on the capacity of interaction with the cytoplasmic membrane [29]. In addition, Scariot et al. [36] also provided strong evidence that oxygenated monoterpenoids (alcohols and aldehydes) such as GE, carvacrol, and thymol exhibited higher antifungal activity than their corresponding hydrocarbons, esters, and cyclic counterparts, indicating that OH− and O− radicals react with cellular components affecting cell permeability and fungal homeostasis. In our case, GE was incorporated to different biopolymer matrixes containing other ingredients such as BW, sucroesters, and glycerol at different concentrations, affecting the interactions of these components with GE and its antifungal performance in different ways, since they can influence the release ability and consequent availability of the antifungal agent used as coating ingredient [30]. Thus, for example, Fernández-Catalán et al. [37] also found differences in the performance of the antifungal GRAS salt potassium bicarbonate (PBC) depending on the coating matrix into which it was incorporated. Whereas it was highly effective when incorporated into an HPMC-oleic acid coating, it was not effective in combination with an HPMC-BW coating, although it was incorporated to the former at a lower concentration. In our case, the interactions between GE and the GA-based coating ingredients might have affected the availability of the OH^−^ and O^−^ radicals, decreasing its effectiveness as antifungal agent.

### 3.2. Effect of Coatings on Plum Fruit Quality

#### 3.2.1. Plum Weight Loss

After 5 and 8 weeks of cold storage at 1 °C, both periods followed by 3 days at 7 °C plus 7 days at 20 °C simulating transport and shelf-life conditions, plum weight loss ranged from 1.2 to 1.3% and from 2.8 to 3.3%, respectively. After both storage periods, no significant differences in weight loss were found among treatments and none of the coatings significantly reduced weight loss with respect to uncoated plums. In general, most edible coatings used for fruits and vegetables are composite coatings based on biopolymers (polysaccharides and/or proteins) as hydrophilic components and lipids as hydrophobic components. The lipidic compounds overcome the hydrophilic character of polar hydrocolloids, conferring both moisture and gas barrier properties to the fruit [38]. However, several works have reported that the moisture barrier of edible coatings formulated with the incorporation of lipids is not always effective, since weight loss does not only depend on the hydrophobic nature of the formulation but also on other coating properties such as mechanical integrity, as well as the characteristics of the fruit [39]. Thus, for example, biopolymer–lipid composite coatings did not reduce weight loss in apples [40], cherry tomatoes [41], table grapes [42], persimmon [43], and oranges [44,45], among others. In the particular case of plums, recent works have reported the inability of coating formulations based on alginate, chitosan, starch, whey protein, pectin, and carboxy methylcellulose to create an effective moisture barrier between the fruit and the environment [13,21,22], while other works have showed a positive effect of similar biopolymer-based coatings reducing plum weight loss [20,24,46]. These works illustrate the amount and complexity of factors that affect the barrier properties of edible coatings for a particular fruit. The wide variety of biopolymers with different molecular weight and degree of esterification/acetylation, hydrophobic ingredients, plasticizers, emulsifiers, and other active ingredients might explain the differences in the results, as they affect the interactions among coating ingredients that determine barrier and mechanical properties, as well as the coating–fruit interactions that determine fruit wettability. Furthermore, factors such as cultivar, physiological stage, and storage conditions play a key role in the performance of the coatings to maintain the fruit postharvest quality. In previous studies conducted at the IVIA, HPMC coatings containing at least 20% BW (*w*/*w*, dry basis) were required to reduce weight loss of ‘Angeleno’ plums, while weight loss increased on plums treated with coatings with above 40% BW content despite increasing coating hydrophobicity, which was attributed to an increase in coating brittleness [39].

#### 3.2.2. Plum Flesh Firmness

**Figure 2:**
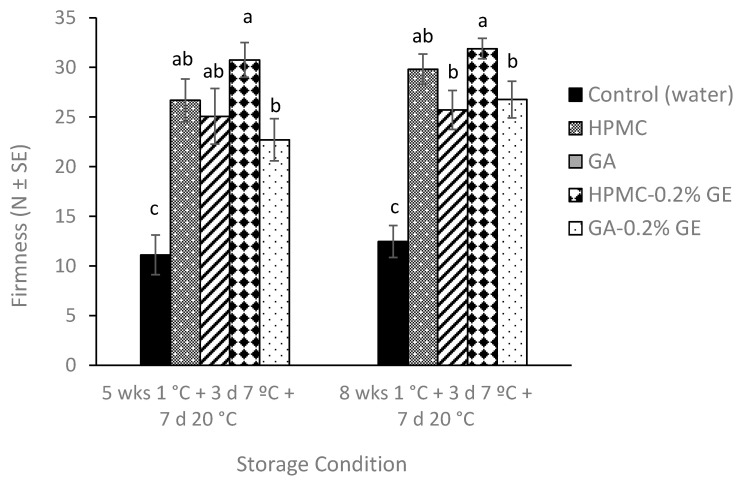
Firmness (N) of ‘Angeleno’ plums uncoated (control) or coated with hydroxypropyl methylcellulose (HPMC)-or gum Arabic (GA)-based edible composite coatings amended with geraniol (GE) after storage for 5 and 8 weeks at 1 °C, followed by 3 days at 7 °C plus 7 days at 20 °C. For each evaluation date, different letters indicate significant differences using Fisher’s protected LSD test (*p* < 0.05). Firmness at harvest was 30.4 ± 1.5 N.

Figure 2 shows firmness of coated and uncoated ‘Angeleno’ plums after 5 and 8 weeks of cold storage at 1 °C, followed by 3 days at 7 °C plus 7 days at 20 °C. At harvest, plums were very firm (30.4 ± 1.5 N), and firmness of uncoated plums significantly decreased after both storage periods to values of 12–14 N, which represents a firmness reduction higher than 50%. In contrast, coated fruit maintained firmness after both storage periods, with values in the range of 25–30 N. In general, no significant differences in fruit firmness were observed between HPMC- and GA-based coatings, although slightly smaller values were obtained for the latter. For each specific biopolymer, the incorporation of GE to the formulation did not affect fruit firmness (*p* > 0.05).

Fruit firmness is one of the main quality attributes considered by stone fruit consumers. Loss of cell wall integrity during storage, caused by loss of water and fruit ripening, leads the fruit to softening, shriveling, and wilting [22,47]. In our work, edible coatings did not control weight loss; therefore, the changes in plum firmness can be mainly attributed to the effect of coating on fruit ripening. As fruit ripens, hydrolyzing enzymes such as polygalacturonase, β-galactosidase, and pectin methyl esterase degrade the main polysaccharides present in the cell wall, contributing to firmness losses [24,46]. It is well reported in the literature that O_2_ reductions and/or CO_2_ increases during storage of climacteric fruit have a positive effect in reducing firmness loss by reducing the respiration rate and the activity of the cell wall-degrading enzymes [21]. Therefore, the highest firmness of coated ‘Angeleno’ plums in this work might be related with the ability of the coatings to provide a semipermeable barrier to O_2_ and CO_2_. Such an effectiveness of GA-and HPMC-based coatings in maintaining firmness of plums has been also reported in other works. For instance, Fawole et al. [22] evaluated the efficacy of GA, gellan gum, chitosan, and alginate coatings formulated with canola oil to maintain the quality of ‘African Delight’ plums and observed that GA was the most effective coating for retaining fruit firmness during 5 weeks of cold storage. Working with ‘Blackamber’ plums, the incorporation of essential oils to the GA biopolymer had a positive effect in reducing firmness loss compared to the GA coating without oil [23]. Similar results were reported for HPMC-based coatings, where the coatings formulated with essential oils provided a better retention of plum firmness than those without essential oils, which correlated with a lower fruit respiration rate and ethylene production [8]. In previous research by our group, HPMC-BW coatings also retained plum firmness during cold storage and shelf life, particularly those with lower BW content, which was related to the ability of these coatings to create a modified atmosphere in the fruit [1,24,39].

#### 3.2.3. Plum Peel Color

Color changes in stone fruits occur as a result of anthocyanin and carotenoid synthesis during maturation and ripening [46]. Changes in pigment contents can be accelerated by stress such as chilling injury, but they can also occur naturally during storage [48]. Table 1 shows the color parameters of coated and uncoated plums after 5 and 8 weeks of storage at 1 °C followed by 3 days at 7 °C plus 7 days at 20 °C. All the color parameters decreased with storage time for coated and uncoated samples, indicating changes towards colors less vivid (lower C*), dark (lower L*), and red (lower hue). In general, all the coating treatments delayed this process with respect to uncoated control samples. At end of the storage period, a*, b*, C*, and hue values decreased by 42, 92, 46, and 95%, respectively, for control samples and an average of 23, 50, 27, and 37%, respectively, for coated samples. Differences between coated and uncoated samples in L* parameter were lower for both storage periods. The incorporation of GE to the HPMC matrix increased the L* values of coated samples compared to the HPMC coating without the essential oil, while no significant differences were observed between GA coatings with and without GE. Nevertheless, at the end of the storage period, all the coated samples showed lower L* values than control samples. When comparing coating matrixes, HPMC-based coatings maintained better the peel color attributes than GA-based coatings. In addition, the incorporation of 0.2% GE to the HPMC coating also resulted in a better maintenance of peel color, while no differences were observed between GA- and GA-0.2% GE-coated plums.

In climacteric fruits such as plums, ripening after harvest is typically driven by an increase in CO_2_ and ethylene production, which triggers changes in quality parameters such as color, texture, and flavor that strongly determine consumer acceptability [49]. The minor changes in color parameters observed in coated samples suggests a delay in ripening associated with the barrier to gases exerted by the coating emulsions and correlates with the behavior observed in the analysis of fruit firmness, with higher values in coated than in control samples, indicating that the coatings effectively modified the plum respiration and ethylene production rates and slowed down the fruit’s metabolism [50]. Similar results have been reported in plums coated with HPMC [8,24], chitosan and alginate [22,46,50], GA [23], and starch/whey protein [13,16]. In addition, some studies have also confirmed that the incorporation of essential oils improves the effect of some polysaccharide coatings, such as aloe vera gel, GA, and HPMC, on delaying plum ripening parameters such as color and firmness [8,17,23,51].

#### 3.2.4. Plum Internal Quality

Soluble solids content (SSC) and titratable acidity (TA) are important factors in determining the quality of stone fruits, since the SSC:TA ratio greatly contributes to fruit taste [52]. In general, chemical changes during postharvest storage of fruits often include a decrease in TA, because organic acids are used as primary substrates for respiration and other metabolic processes, and an increase in SSC, as starch is broken into simple sugar molecules [53]. In this work, TA, SSC, and MI of ‘Angeleno’ plums at harvest were 8.1 ± 0.2 g/L (malic acid), 18.7 ± 0.1 °Brix, and 23.2 ± 0.7, respectively. TA and SSC values significantly decreased, and MI increased during storage for both coated and control fruit. After storage, TA was not affected by coating application, while SSC varied between 16.2 and 17.4% (Table 2). The effect of the coatings on SSC depended on the storage condition, which makes it difficult to draw any conclusion regarding the effect of the coatings on this quality parameter. Thus, after 5 weeks of cold storage, only plums coated with the HPMC coating (without GE) had higher SSC than uncoated plums, whereas at the end of the storage period (8 weeks plus transportation and shelf life), all the coated plums, except for those coated with HPMC-0.2% GE, had higher SSC than uncoated plums.

In general, plum internal quality parameters can be affected by coating type, fruit type and cultivar, and storage conditions. Some authors have reported an effect of coating application on the maintenance of both SSC and TA of plums during storage, while others found an effect on TA but not on SSC or vice versa [8,22,24,46,54]. This variability in the behavior of coated plums can be attributed not only to the composition of the coating but also to physiological aspects related to cultivar, ripening state at harvest, and storage conditions. Some workers have reported that in climacteric fruits, ripening-associated traits such as SSC and TA are less dependent on the action of ethylene than firmness or color changes [55,56]. This could explain why, in this work, HPMC and GA coatings contributed to maintain fruit firmness and color, while they did not affect the parameters that determine the fruit internal maturity.

#### 3.2.5. Ethanol and Acetaldehyde Contents

The resistance to O_2_ and CO_2_ diffusion created by coating application to fresh fruit can lead to lower fruit respiration and ethylene production and can, therefore, have a positive impact on some physical properties of the fruit, such as reduced softening or color change, as described above [21]. This gas barrier usually leads to an increase in the content of the fermentation volatiles ethanol and acetaldehyde, which is influenced by the properties of the coating emulsion, the coated horticultural product, and the postharvest storage conditions (mainly temperature and length). In this research, the volatile contents of coated ‘Angeleno’ plums were significantly affected by coating application and storage time (Table 3). For both storage periods, the ethanol of the HPMC-coated plums was significantly higher than that of the uncoated plums, while no significant differences were observed between the control and the GA-coated plums. On the other hand, the incorporation of GE to the HPMC-based coating increased the ethanol level in coated plums for both storage periods. In the case of acetaldehyde, its content increased with storage time. However, although significant differences were observed among treatments, no increase was observed due to coating application except in plums coated with HPMC-0.2% GE at the end of storage. These results confirm that HPMC-based coatings exerted a higher gas barrier than GA-based coatings, inducing a higher content of ethanol in plums.

Several reviews have described the good gas barrier properties of biopolymers such as HPMC and GA in combination with different hydrophobic compounds and their effect on the preservation of fruits and vegetables [57,58,59,60]. However, the gas barrier of these coatings when applied to fruits and vegetables is greatly affected by many factors, such as interactions between the polymer matrix and other coating ingredients, the coating–fruit interaction, the coating solid content and viscosity, and the fruit physiological state. Thus, HPMC-based coatings formulated with different hydrophobic compounds significantly increased the levels of ethanol and acetaldehyde in different plum cultivars after cold and shelf-life storage [1,24,61], and the HPMC content was the factor that mostly influenced the levels of these volatiles. In the case of GA-based coatings, although several works have reported an influence of coating on plum quality traits such as firmness and maturity index associated with the gas barrier of the coatings, no references have been found regarding ethanol and acetaldehyde content [23,52]. In a study with ‘African Delight’ plums, volatile analysis of fruit coated with different biopolymers, including a GA-based coating, showed no significant differences in the levels of the fermentation esters screened between treatments throughout storage [22]. On the other hand, the results of our work showed that adding GE to the HPMC-based coating increased ethanol and acetaldehyde contents of coated plums by 96 and 24%, respectively, compared to uncoated plums at the end of the storage (8 weeks plus transportation and shelf life). The addition of different essential oils to HPMC stand-alone films was reported to reduce the O_2_ permeability of the films, although the effect depended on the essential oil. Thus, clove, sage, and oregano essential oils improved the O_2_ barrier of HPMC films, which was attributed to the essential oils filling the empty spaces of the HPMC matrix [62], whereas fingerroot and plai essential oils increased O_2_ permeability [63].

#### 3.2.6. Plum Physiological Disorders

The use of low temperatures during storage greatly increases the storability and market life of plums. Temperatures around 0 °C are generally the most suitable for long-term storage of stone fruits [31]. However, most plum cultivars are highly susceptible to chilling injury if cold storage conditions and/or duration are not appropriate. The most important chilling injury symptoms on plums are gel breakdown, flesh browning, translucency, and flesh bleeding [64]. These symptoms develop mainly when fruit are transferred to 20 °C after cold storage and their intensity increases when fruit have been stored at temperatures between 2.2 and 7.6 °C. Therefore, from this point of view, a storage temperature of 0–1 °C is considered as suitable to minimize chilling injury problems [31]. In the present work with ‘Angeleno’ plums, a storage period of 3 days at 7 °C was simulated after cold storage at 1 °C and before shelf life at 20 °C because this temperature is reached very often during transportation and is within the so-called ‘killing temperature zone’ in terms of plum physiological disorders [31]. The main chilling injury symptom observed in this work at the end of both storage periods was flesh bleeding. This disorder manifests with the accumulation of anthocyanins either around the stone or immediately beneath the epidermis, where the pigments are originally located, and their diffusion throughout the plum flesh, causing an intense red color. Early research showed that plum sensitivity to chilling injury is probably related to an increase in the rate of ripening, suggesting the putative role of ethylene [3,65] and the benefits of controlled atmosphere storage as an effective preventive means [66]. In addition, the cell wall degradation that leads to plum flesh softening may enhance the diffusion of anthocyanins and hence the incidence of bleeding [61]. Table 4 shows flesh bleeding in coated and uncoated ‘Angeleno’ plums after 5 and 8 weeks of cold storage followed by transport and shelf life. After both storage periods, the coatings were effective in reducing plum flesh bleeding when compared to uncoated controls. Flesh bleeding of uncoated samples was between moderate and severe after 5 weeks of cold storage, but it was severe (more than 50% of affected flesh) after 8 weeks. Both coatings significantly reduced chilling injury, and HPMC-based coatings were more effective than GA-based coatings. No significant differences were observed between coatings formulated with or without GE. Similar results have been reported for ‘Friar’ [24], ‘Autumn Giant’, and ‘Angeleno’ plums [39,61] coated with other HPMC-based coatings. These results were attributed to the modified atmosphere created in the fruit by the coatings. Thus, flesh bleeding was inversely correlated with high plum firmness and ethanol and acetaldehyde content in the juice, which can be associated with the gas barrier provided by the coatings [67]. In our work, HPMC coatings maintained the firmness and color of plums better than GA coatings and the obtained levels of ethanol and acetaldehyde confirm their greater gas barrier effect.

#### 3.2.7. Plum Sensory Quality

**Figure 3:**
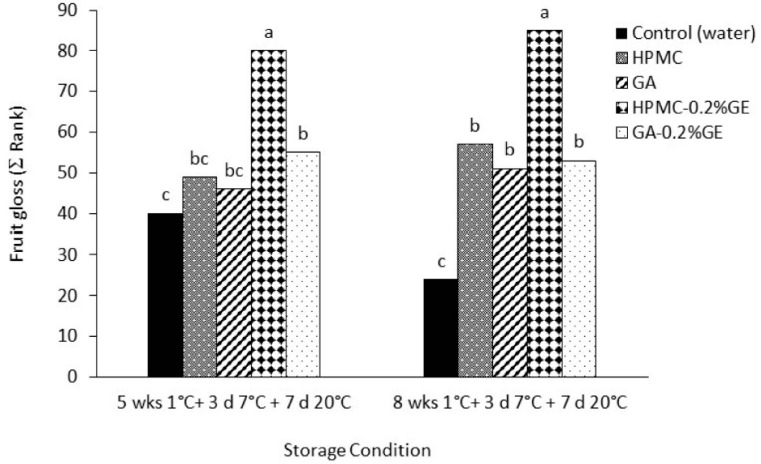
Visual gloss (rank summation of 18 panelists from 1 (least glossy treatment) to 5 (glossiest treatment)) of ‘Angeleno’ plums uncoated (control) or coated with hydroxypropyl methylcellulose (HPMC)-or gum Arabic (GA)-based edible composite coatings amended with geraniol (GE) and stored for 5 and 8 weeks at 1 °C, followed by 3 days at 7 °C plus 7 days at 20 °C. For each evaluation date, different letters indicate significant differences using the Friedman test (*p* < 0.05).

Sensory quality evaluations of ‘Angeleno’ plums are presented in Table 5. At harvest, plums were evaluated as very firm at the touch of the fruit (4.6 ± 0.5; 1–5 scale) and with an acceptable overall flavor (5.2 ± 0.5, 1–9 scale). Coating application, irrespective of GE addition, did not result in significant differences in overall flavor and off-flavor after both 5- and 8-week storage periods. These results indicate that the accumulation of ethanol and acetaldehyde in HPMC-coated plums after storage was below the detection threshold for this cultivar and did not negatively affect these sensory attributes. In the case of firmness, coated plums retained a higher firmness than uncoated controls at the end of storage, in agreement with the instrumental firmness analysis. On the other hand, edible coating application improved fruit gloss, with the HPMC-0.2% GE coating providing the highest gloss (Figure 3). This is a significant result since fruit gloss is an important quality trait for the marketability of plums.

## 4. Conclusions

The primary results of this work show that HPMC-based coatings containing the EO GE at a concentration of 0.2% have the potential to reduce brown rot incidence and severity on ‘Angeleno’ plums during long-term cold storage, while the same concentration of GE incorporated to GA-based coatings did not affect disease development. Furthermore, HPMC and GA coatings contributed to maintaining fruit firmness and color, while they did not affect the internal maturity and sensorial quality attributes. When comparing coating matrixes, HPMC-based coatings maintained better fruit color and firmness and significantly reduced chilling injury symptoms manifested as flesh bleeding, even after a simulated storage period of 3 days at 7 °C after cold storage at 1 °C and before shelf life at 20 °C, which is within the so-called ‘killing temperature zone’ in terms of plum physiological disorders. These results correlate with the higher gas barrier of the HPMC-based coatings compared to the GA-based coatings. According to these results, the HPMC-based coating formulated with 0.2% GE shows potential to be used as a nonpolluting alternative for extending plum shelf life, although further research should focus on improving the moisture barrier of the coating.

## Figures and Tables

**Table 1:** Values of color attributes (CIELAB parameters) of ‘Angeleno’ plums uncoated (control) or coated with hydroxypropyl methylcellulose (HPMC)- or gum Arabic (AG)-based edible composite coatings amended with geraniol (GE) after storage for 5 and 8 weeks at 1 °C followed by 3 days at 7 °C plus 7 days at 20 °C.

Storage Period at 1 °C	Treatment	L*	a*	b*	C*	Hue
5 weeks	Control	27.5 ± 0.4 ^b,c^	11.0 ± 1.0 ^d^	1.4 ± 0.5 ^d^	11.2± 1.1 ^d^	2.7 ± 2.5 ^d^
	HPMC	26.7 ± 0.4 ^c^	18.1 ± 1.0 ^b^	6.3 ± 0.7 ^b^	19.3± 1.2 ^b^	17.3 ± 1.3 ^a,b^
	GA	27.7 ± 0.4 ^a,b,c^	14.7 ± 0.9 ^c^	3.3 ± 0.5 ^c^	15.1± 1.0 ^c^	11.0 ± 1.3 ^c^
	HPMC-0.2% GE	28.7 ± 0.5 ^a^	22.6 ± 0.8 ^a^	9.0 ± 0.6 ^a^	24.4± 1.0 ^a^	21.3 ± 0.7 ^a^
	GA-0.2% GE	27.9 ± 0.4 ^a,b^	16.8 ± 1.1 ^b,c^	5.1 ± 0.6 ^b^	17.6± 1.2 ^b,c^	15.0 ± 1.3 ^b,c^
8 weeks	Control	28.5 ± 0.4 ^a^	11.9 ± 0.8 ^c^	0.7 ± 0.4 ^c^	12.1 ± 0.8 ^c^	1.0 ± 2.2 ^d^
	HPMC	24.9 ± 0.4 ^c^	15.6 ± 1.0 ^b^	4.3 ± 0.5 ^b^	16.2 ± 1.1 ^b^	14.4 ± 0.8 ^a,b^
	GA	27.3 ± 0.4 ^b^	14.6 ±1.1 ^b^	3.2 ± 0.7 ^b^	15.0 ± 1.3 ^b^	9.8 ± 1.5 ^c^
	HPMC-0.2% GE	26.9 ± 0.3 ^b^	19.5 ± 0.7 ^a^	6.5 ± 0.5 ^a^	20.6 ± 0.9 ^a^	17.8 ± 0.9 ^a^
	GA-0.2% GE	26.3 ± 0.3 ^b^	14.1 ±0.7 ^b,c^	3.0 ± 0.3 ^b^	14.5 ± 0.7 ^b,c^	11.5 ± 0.8 ^b,c^

Values at harvest: L* = 33.7 ± 3.2; a* = 20.8 ± 0.8; b* = 8.6 ± 0.8; C* = 22.7 ± 1.0; hue = 21.5 ± 1.4. Values are means ± SE. For each color parameter and evaluation date, different letters indicate significant differences using Fisher’s protected LSD test (*p* < 0.05).

**Table 2:** Juice quality attributes of ‘Angeleno’ plums uncoated (control) or coated with hydroxypropyl methylcellulose (HPMC)- or gum Arabic (GA)-based edible composite coatings amended with geraniol (GE) after storage for 5 and 8 weeks at 1 °C, followed by 3 days at 7 °C plus 7 days at 20 °C.

Storage Period at 1 °C	Treatment	TA (g/L Malic Acid)	SSC (°Brix)	MI
5 weeks	Control	6.6 ± 0.4 ^a^	16.9 ± 0.1 ^b^	25.6 ± 0.7 ^a^
	HPMC	7.0 ± 0.5 ^a^	17.4 ± 0.0 ^a^	25.1 ± 0.7 ^a^
	GA	6.6 ± 0.2 ^a^	16.7 ± 0.3 ^b^	25.2 ± 0.3 ^a^
	HPMC-0.2% GE	6.9 ± 0.6 ^a^	17.1 ± 0.1 ^a,b^	24.9 ± 0.8 ^a^
	GA-0.2% GE	7.0 ± 0.6 ^a^	16.2 ± 0.2 ^c^	23.2 ± 0.6 ^b^
8 weeks	Control	6.6 ± 0.2 ^a^	16.5 ± 0.2 ^b^	25.1 ± 0.5 ^b^
	HPMC	7.1 ± 0.4 ^a^	17.1 ± 0.1 ^a^	24.0 ± 0.5 ^b^
	GA	6.8 ± 0.4 ^a^	17.4 ± 0.3 ^a^	25.5 ± 0.4 ^b^
	HPMC-0.2% GE	6.5 ± 0.6 ^a^	16.3 ± 0.2 ^b^	25.0 ± 0.9 ^b^
	GA-0.2% GE	6.1 ± 0.2 ^a^	17.3 ± 0.1 ^a^	28.2 ± 0.3 ^a^

Values at harvest: titratable acidity (TA) = 8.1 ± 0.2 g/L (malic acid); soluble solid content (SSC) = 18.7 ± 0.1 °Brix; and maturity index (MI = SSC/TA) = 23.2 ± 0.7 (calculated with TA as %). Values are means ± SE. For each quality attribute and evaluation date, different letters indicate significant differences using Fisher’s protected LSD test (*p* < 0.05).

**Table 3:** Contents of ethanol and acetaldehyde in ‘Angeleno’ plums uncoated (control) or coated with hydroxypropyl methylcellulose (HPMC)- or gum Arabic (GA)-based edible composite coatings amended with geraniol (GE) and stored for 5 and 8 weeks at 1 °C, followed by 3 days at 7 °C plus 7 days at 20 °C.

Storage Period at 1 °C	Treatment	Ethanol (mg/L)	Acetaldehyde (mg/L)
5 weeks	Control	13.5 ± 7.3 ^c^	5.7 ± 1.2 ^a^
	HPMC	51.3 ± 3.7 ^b^	4.0 ± 0.3 ^b,c^
	GA	3.1 ± 0.2 ^c^	3.0 ± 0.6 ^c^
	HPMC-0.2% GE	94.0 ± 19.5 ^a^	5.2 ± 0.9 ^a,b^
	GA-0.2% GE	16.6 ± 5.9 ^c^	3.8 ± 0.7 ^b,c^
8 weeks	Control	21.3 ± 6.9 ^c^	15.8 ± 1.2 ^b^
	HPMC	212.7 ± 91.8 ^b^	12.5 ± 2.2 ^c^
	GA	15.4 ± 1.2 ^c^	8.9 ± 2.0 ^d^
	HPMC-0.2% GE	587.7± 44.5 ^a^	20.7 ± 1.4 ^a^
	GA-0.2% GE	46.2 ± 14.1 ^c^	6.7± 0.8 ^d^

Values at harvest: ethanol content = 31.7 ± 12.5 mg/L; acetaldehyde content = 2.9 ± 0.4 mg/L. Values are means ± SE. For each volatile and evaluation date, different letters indicate significant differences using Fisher’s protected LSD test (*p* < 0.05).

**Table 4:** Flesh bleeding of ‘Angeleno’ plums uncoated (control) or coated with hydroxypropyl methylcellulose (HPMC)- or gum Arabic (GA)-based edible composite coatings amended with geraniol (GE) after 5 and 8 weeks of storage at 1 °C, followed by 3 days at 7 °C plus 7 days at 20 °C.

Treatment	Storage Conditions	
	5 Weeks 1 °C + 3 d 7 °C + 7 d 20 °C	8 Weeks 1 °C + 3 d 7 °C + 7 d 20 °C
Control	2.4 ± 0.1 ^a^	3.0 ± 0.0 ^a^
HPMC	1.9 ± 0.2 ^b^	1.9 ± 0.2 ^c^
GA	2.1 ± 0.2 ^a,b^	2.7 ± 0.1 ^a,b^
HPMC-0.2% GE	1.5 ± 0.1 ^c^	1.9 ± 0.2 ^c^
GA-0.2% GE	2.0 ± 0.2 ^b^	2.6 ± 0.1 ^b^

Plum flesh bleeding rated with scores from 1 (none) to 3 (severe, >50% of the flesh surface). For each evaluation date, different letters indicate significant differences using Fisher’s protected LSD test (*p* < 0.05). Values are means ± SE.

**Table 5:** Overall flavor, off-flavor, firmness, and juiciness of ‘Angeleno’ plums uncoated (control) or coated with hydroxypropyl methylcellulose (HPMC)- or gum Arabic (GA)-based edible composite coatings amended with geraniol (GE) after storage for 5 and 8 weeks at 1 °C, followed by 3 days at 7 °C plus 7 days at 20 °C.

Storage Period at 1 °C	Treatment	Overall Flavor	Off-Flavor	Firmness
5 weeks	Control	6.3 ± 0.4 ^a^	1.1 ± 0.1 ^a^	2.5 ± 0.2 ^b^
	HPMC	5.6 ± 0.3 ^a^	1.4 ± 0.1 ^a^	3.3 ± 0.3 ^a^
	GA	5.8 ± 0.4 ^a^	1.3 ± 0.2 ^a^	3.7 ± 0.2 ^a^
	HPMC-0.2% GE	5.8 ± 0.4 ^a^	1.3 ± 0.2 ^a^	3.8 ± 0.2 ^a^
	GA-0.2% GE	6.0 ± 0.5 ^a^	1.1 ± 0.1 ^a^	3.6 ± 0.2 ^a^
8 weeks	Control	4.2 ± 0.5 ^a^	1.0 ± 0.1 ^a^	2.8 ± 0.2 ^b^
	HPMC	4.3 ± 0.3 ^a^	1.0 ± 0.1 ^a^	3.3 ± 0.2 ^a^
	GA	4.3 ± 0.4 ^a^	1.0 ± 0.2 ^a^	3.3 ± 0.2 ^a^
	HPMC-0.2% GE	4.5 ± 0.4 ^a^	1.0 ± 0.2 ^a^	3.5 ± 0.2 ^a^
	GA-0.2% GE	5.0 ± 0.4 ^a^	1.0 ± 0.0 ^a^	4.0 ± 0.2 ^a^

Values at harvest: overall flavor = 5.2 ± 0.5; off-flavor = 1.1 ± 0.1; firmness = 4.6 ± 0.2. Values are means ± SE. Overall flavor rated with scores from 1 (very poor) to 9 (optimum), off-flavor from 1 (absence) to 5 (high presence), and firmness from 1 (very soft) to 5 (very firm). For each sensory attribute evaluation date, different letters indicate significant differences using Fisher’s protected LSD test (*p* < 0.05).

## Data Availability

The data presented in this study belong to the IVIA and are available upon request from the corresponding author.
